# Comparison of weight per volume and protein nitrogen units in non-standardized allergen extracts: implications for prescribing subcutaneous immunotherapy

**DOI:** 10.1186/s13223-021-00588-5

**Published:** 2021-09-16

**Authors:** Benny Dua, Jane Park, Harold Kim

**Affiliations:** 1 Division of Clinical Immunology and Allergy, Department of Medicine, McMaster University, Hamilton, ON Canada; 2 Department of Medicine, Western University, London, ON Canada; 3 Division of Clinical Immunology and Allergy, Department of Medicine, Western University, London, ON Canada

**Keywords:** Non-standardized extract, PNU, Weight per volume

## Abstract

**Background:**

Allergen extracts used in subcutaneous immunotherapy can be standardized or non-standardized. Standardized extracts are available in specific biological potencies, presumably making their biological activity more consistent. The majority of allergen extracts are non-standardized and may have less consistent potencies. Non-standardized extracts are labeled as weight per volume or protein nitrogen units (PNUs). Neither method provides direct information regarding the extract’s biologic potency. The purpose of this study was to compare weight per volume versus PNU concentrations for 4 non-standardized allergen extracts prepared by two allergen manufacturers. The potencies were compared to current North American practice recommendations.

**Methods:**

The weight per volume and PNU values were provided for 4 non-standardized extracts—birch, short ragweed, dog hair and Alternaria—from HollisterStier and Stallergenes Greer. Weight per volume and PNU concentrations were compared for each of these extracts from both manufacturers. From the raw data, we calculated the corresponding PNU values for a weight per volume of 1:100 and 1:200 for each extract. Similarly, we calculated the corresponding weight per volume including a range of PNU values, for 1000, 2000, 3000, 4000 and 5000 PNU/ml.

**Results:**

Birch extract has low PNU concentration, below 5000, for a weight per volume of 1:200 for both HollisterStier and Stallergenes Greer. In contrast, for both HollisterStier and Stallergenes Greer ragweed extract, a weight per volume of 1:200 corresponds to a PNU concentration greater than 5000. Dog extract for a weight per volume of 1:200, and even for 1:100, corresponds to very low PNUs for both companies. For Alternaria, corresponding PNU concentrations for HollisterStier is low at only 500 while over 5000 for Stallergenes Greer.

**Conclusions:**

Our results show variability when comparing weight per volume and PNU concentrations for both Hollister-Stier and Stallergenes Greer products. We suggest selecting a PNU dose that corresponds to a weight per volume of 1:200 as this may improve patient safety. Our recommendations for starting PNU dose for the four non-standardized extracts are 1500 for birch, 5000 for ragweed, 25 for dog, and 500 for Alternaria when using HollisterStier products; 2300 for birch, 5000 for ragweed, 1200 for dog, and 5000 for Alternaria when using Stallergenes Greer products. If the starting PNU concentration is considerably below 5000 for a weight per volume of 1:200 slow up-titration is advised. Conversely, for PNU concentrations above 5000 for weight per volume of 1:200 we suggest a maintenance dose of 5000 PNU.

## Background/Introduction

Allergen immunotherapy is used for the treatment of allergic rhinitis and asthma [1–6]. Many patients with allergic rhinitis and asthma are inadequately controlled with appropriate allergen avoidance and medical therapy; thus, immunotherapy is a desired and effective treatment modality for these patients [7, 8]. Double-blind placebo-controlled studies have proven the clinical effectiveness of immunotherapy in both adults and children with allergic rhinitis and asthma [1–6]. Furthermore, allergen immunotherapy may be disease modifying and may reduce the risk of future development of asthma as well as improve quality of life in patients with allergic rhinitis [8].

Allergen extracts used in immunotherapy can be either standardized or non-standardized. Standardized extracts are available in specific biological potencies, typically expressed in units of BAU, AU or micrograms of allergen, depending on the extract. However, depending on the manufacturer, alternative allergen units for biological potencies have been reported such as index of reactivity (IR) and index of concentration (IC). The advantage of standardized extracts is that the biological activity is more consistent. This may improve the likelihood of efficacy and minimize the risk of an adverse reaction [8]. Despite this level of quality control for standardized extracts, the majority of clinically used allergen extracts are non-standardized and consequently, may have less consistent potencies. Non-standardized extracts can be labelled as weight per volume, which expresses weight in grams per volume in milliliters. For example, weight per volume of 1:100 potency implies that 1 g of dry allergen was added to 100 mL of an extraction buffer [8]. Alternatively, these extracts can also be labeled in protein nitrogen units (PNUs), where 1 PNU is equal to 0.01 g of protein nitrogen per milliliter [8]. PNU allows for quantitative measurement of the protein in nitrogen units, which in theory should correlate better with appropriate antibody response during immunotherapy. Nonetheless, neither method allows for any direct or comparative information regarding the extract’s biologic potency and should not be considered equipotent [8]. Therefore, the variability in potencies of non-standardized extracts may have important clinical consequences, particularly in the treatment with allergen immunotherapy.

There is a paucity of data with respect to comparing these two units of extract quantification for clinical use. The American Academy of Allergy, Asthma, and Immunology (AAAAI) has recommended using potencies of 1:100 to 1:200 weight per volume or 3000 to 5000 PNU, both at a volume of 0.5 ml as maintenance doses [8]. In an attempt to simplify prescribing, the CSACI has recommended a dose of 5000 PNU/ml at a volume of 0.5 ml as maintenance doses [7]. As such, the purpose of this study was to compare the weight per volume and PNU concentrations for 4 non-standardized extracts (birch, ragweed, dog and Alternaria) prepared by two manufacturers, and evaluate how these potencies compare to current practice recommendations with respect to allergen immunotherapy dosing. Moreover, in order to emphasize safety with prescribing, we will suggest prescribing doses in safest PNU that correspond to the weakest weight per volume recommendations by AAAAI.

## Methods

Data was provided to us from HollisterStier and Stallergenes Greer, two of the three major allergen manufacturers available in Canada for this study. The weight per volume and PNU values were provided for 4 non-standardized extracts including birch mix, short ragweed, dog hair and Alternaria from each company. For HollisterStier, the extract data was retrieved from 1 lot, containing 50% glycerin and manufactured in 2018–2019. For Stallergenes Greer, the extract data was retrieved from 5 consecutive lots, containing no glycerin and manufactured from 2008 to 2013.

Source materials used in the manufacturing of allergen extracts were collected from natural sources or from laboratory cultures. The extracts were labelled as weight per volume based on the weight of the source material to the volume of the extracting fluid. The weight per volume concentrations for the aforementioned extracts were all labelled as either 1:10 or 1:20.

PNU was measured using the Kjeldahl method. In brief, the protein in the allergic sample is precipitated with 1 mL of phosphotungstic acid (PTA), which separates the protein nitrogen from other nitrogen containing constituents in the sample. The sample is then injected into a high temperature furnace where it is catalytically combusted at approximately 850 °C. Oxidative pyrolysis causes the chemically bound nitrogen to be converted to nitric oxide. A flow of oxygen transports the nitric oxide to the chemiluminescence detector where the nitrogen concentration is determined. One PNU per mL is equivalent to 1 × 10^–5^ mg nitrogen. PNU values for the aforementioned extracts were provided and the range of PNUs varied according to the specific allergen (birch mix, short ragweed, dog hair and Alternaria).

Weight per volume and PNU concentrations were compared for each of the 4 non-standardized extracts from both manufacturers. From the raw data, we calculated the corresponding PNU values for a weight per volume of 1:100 and 1:200 for each extract. Similarly, we calculated the corresponding weight per volume for a range of PNU values, for 1000, 2000, 3000, 4000 and 5000 PNU/ml. These calculations were based on simple proportion ratios from the raw data. These measurements were used as they correspond to the recommended doses from the AAAAI and CSACI [7, 8].

## Results

### Birch Mix

For birch mix, HollisterStier provided 13 extract concentrations at 1:20 weight per volume, with corresponding PNU values ranging from 14,500 to 22,000. Stallergenes Greer provided 5 extract concentrations at 1:10 weight per volume, with PNU values ranging from 45,000 to 57,000.

Table 1A, B illustrate the corresponding PNU and weight per volume values across a range of birch mix concentrations. For HollisterStier, a weight per volume of 1:100 corresponds to a mean PNU of 3569.23, and a weight per volume of 1:200 corresponds to a mean PNU of 1784.62. Conversely, a PNU concentration of 3000 corresponds to a mean weight per volume of 1:119, and a PNU concentration of 5000 corresponds to a mean weight per volume of 1:71. For Stallergenes Greer, a weight per volume of 1:100 corresponds to a mean PNU of 5100, and a weight per volume of 1:200 corresponds to a mean PNU of 2550.00. Conversely, a PNU concentration of 3000 corresponds to a mean weight per volume of 1:170, and a PNU concentration of 5000 corresponds to a mean weight per volume of 1:102.

**Table 1:** Birch extract data from HollisterStier and Stallergenes Greer

BIRCH	Corresponding PNU/mL			
	HollisterStier (n = 13)		Stallergenes Greer (n = 5)	
	Wt/volume (1:100)	Wt/volume (1:200)	Wt/volume (1:100)	Wt/volume (1:200)
A				
Lowest value	2900.00	1450.00	4500.00	2250.00
Highest value	4400.00	2200.00	5700.00	2850.00
Mean	3569.23	1784.62	5100.00	2550.00
St. deviation	519.44	259.72	477.49	238.75

BIRCH	Corresponding Diluent (mL)									
	HollisterStier (n = 13)					Stallergenes Greer (n = 5)				
	PNU (1000)	PNU (2000)	PNU (3000)	PNU (4000)	PNU (5000)	PNU (1000)	PNU (2000)	PNU (3000)	PNU (4000)	PNU (5000)
B										
Lowest value	290.00	145.00	96.67	72.50	58.00	450.00	225.00	150.00	112.50	90.00
Highest value	440.00	220.00	146.67	110.00	88.00	570.00	285.00	190.00	142.50	114.00
Mean	356.92	178.46	118.97	89.23	71.38	510.00	255.00	170.00	127.50	102.00
St. deviation	51.94	25.97	17.31	12.99	10.39	47.75	23.87	15.92	11.94	9.55

Corresponding PNU concentrations for a weight per volume of 1:100 and 1:200 are shown A, while corresponding diluent volumes for PNUs values from 1000 to 5000 are shown in B

These volumes represent the amount of diluent or buffer added to 1 g of allergen

### Short Ragweed

For short ragweed, HollisterStier provided 16 extract concentrations at 1:20 weight per volume, with corresponding PNU values ranging from 74,000 to 117,500. Stallergenes Greer provided 5 extract concentrations also at 1:10 weight per volume, with PNU values ranging from 75,000 to 90,000.

Table 2A, B illustrate the corresponding PNU and weight per volume values across a range of ragweed concentrations. For HollisterStier, a weight per volume of 1:100 corresponds to a mean PNU of 17,443.75, and a weight per volume of 1:200 corresponds to a mean PNU of 8721.88. Conversely, a PNU concentration of 3000 corresponds to a mean weight per volume of 1:581, and a PNU concentration of 5000 corresponds to a mean weight per volume of 1:350. For Stallergenes Greer, a weight per volume of 1:100 corresponds to a mean PNU of 16,360, and a weight per volume of 1:200 corresponds to a mean PNU of 8180.00. Conversely, a PNU concentration of 3000 corresponds to a mean weight per volume of 1:545, and a PNU concentration of 5000 corresponds to a mean weight per volume of 1:327.

**Table 2:** Ragweed extract data from HollisterStier and Stallergenes Greer

RAGWEED	Corresponding PNU/mL			
	HollisterStier (n = 16)		Stallergenes Greer (n = 5)	
	Wt/volume (1:100)	Wt/volume (1:200)	Wt/volume (1:100)	Wt/volume (1:200)
A				
Lowest value	14,800.00	7400.00	15,000.00	7500.00
Highest value	23,500.00	11,750.00	18,000.00	9000.00
Mean	17,443.75	8721.88	16,360.00	8180.00
St. deviation	2593.98	1296.99	1209.30	604.65

RAGWEED	Corresponding diluent (mL)									
	HollisterStier (n = 16)					Stallergenes Greer (n = 5)				
	PNU (1000)	PNU (2000)	PNU (3000)	PNU (4000)	PNU (5000)	PNU (1000)	PNU (2000)	PNU (3000)	PNU (4000)	PNU (5000)
B										
Lowest value	1480.00	740.00	493.33	370.00	296.00	1500.00	750.00	500.00	375.00	300.00
Highest Value	2350.00	1175.00	783.33	587.50	470.00	1800.00	900.00	600.00	450.00	360.00
Mean	1744.38	872.19	581.46	436.09	348.88	1636.00	818.00	545.33	409.00	327.20
St. deviation	259.40	129.70	86.47	64.85	51.88	120.93	60.46	40.31	30.23	24.19

Corresponding PNU concentrations for a weight per volume of 1:100 and 1:200 are shown A, while corresponding diluent volumes for PNUs values from 1000 to 5000 are shown in B. These volumes represent the amount of diluent or buffer added to 1 g of allergen

### Dog Hair

For dog hair, HollisterStier provided 12 extract concentrations at 1:10 weight per volume, with corresponding PNU values ranging from 500 to 3000. Stallergenes Greer provided 5 extract concentrations also at 1:10 weight per volume, with PNU values ranging from 24,000 to 37,000.

Table 3A, B illustrate the corresponding PNU and weight per volume values across a range of dog concentrations. For HollisterStier, a weight per volume of 1:100 corresponds to a mean PNU of 208.33, and a weight per volume of 1:200 corresponds to a mean PNU of 104.17. Conversely, a PNU concentration of 3000 corresponds to mean of 1:7, and a PNU concentration of 5000 corresponds to a mean weight per volume of 1:4. For Stallergenes Greer, a weight per volume of 1:100 corresponds to a mean PNU of 3260.00, and a weight per volume of 1:200 corresponds to a mean PNU of 1630. Conversely, a PNU concentration of 3000 corresponds to a mean weight per volume of 1:109, and a PNU concentration of 5000 corresponds to a mean weight per volume of 1:65.

**Table 3:** Dog extract data from HollisterStier and Stallergenes Greer

DOG	Corresponding PNU/mL			
	HollisterStier (n = 12)		Stallergenes Greer (n = 5)	
	Wt/volume (1:100)	Wt/volume (1:200)	Wt/volume (1:100)	Wt/volume (1:200)
A				
Lowest value	50.00	25.00	2400.00	1200.00
Highest value	300.00	150.00	3700.00	1850.00
Mean	208.33	104.17	3260.00	1630.00
St. deviation	73.12	36.56	449.89	224.94

DOG	Corresponding diluent (mL)									
	HollisterStier (n = 12)					Stallergenes Greer (n = 5)				
	PNU (1000)	PNU (2000)	PNU (3000)	PNU (4000)	PNU (5000)	PNU (1000)	PNU (2000)	PNU (3000)	PNU (4000)	PNU (5000)
B										
Lowest value	5.00	2.50	1.67	1.25	1.00	240.00	120.00	80.00	60.00	48.00
Highest value	30.00	15.00	10.00	7.50	6.00	370.00	185.00	123.33	92.50	74.00
Mean	20.83	10.42	6.94	5.21	4.17	326.00	163.00	108.67	81.50	65.20
St. deviation	7.31	3.66	2.44	1.83	1.46	44.99	22.49	15.00	11.25	9.00

Corresponding PNU concentrations for a weight per volume of 1:100 and 1:200 are shown A, while corresponding diluent volumes for PNUs values from 1000 to 5000 are shown in B. These volumes represent the amount of diluent or buffer added to 1 g of allergen

### Alternaria

For Alternaria, HollisterStier provided 27 extract concentrations at 1:10 weight per volume, with corresponding PNU values ranging from 9000 to 41,000. Stallergenes Greer provided 5 extract concentrations at 1:20 weight per volume, with PNU values ranging from 57,000 to 79,000.

Table 4A, B illustrate the corresponding PNU and weight per volume values across a range of Alternaria concentrations. For HollisterStier, a weight per volume of 1:100 corresponds to a mean PNU of 1974.07, and a weight per volume of 1:200 corresponds to a mean of PNU of 987.04. Conversely, a PNU concentration of 3000 corresponds to a mean weight per volume of 1:66, and a PNU concentration of 5000 corresponds to a mean of 1:39. For Stallergenes Greer, a weight per volume of 1:100 corresponds to a mean PNU of 13,480, and a weight per volume of 1:200 corresponds to a mean PNU of 6740.00. Conversely, a PNU concentration of 3000 corresponds to a mean of 1:449, and a PNU concentration of 5000 corresponds to an mean weight per volume of 1:270.

**Table 4:** Alternaria extract data from HollisterStier and Stallergenes Greer

ALT	Corresponding PNU/mL			
	HollisterStier (n = 27)		Stallergenes Greer (n = 5)	
	Wt/volume (1:100)	Wt/volume (1:200)	Wt/volume (1:100)	Wt/volume (1:200)
A				
Lowest value	900.00	450.00	11,400.00	5700.00
Highest value	4100.00	2050.00	15,800.00	7900.00
Mean	1974.07	987.04	13,480.00	6740.00
St. deviation	662.68	331.34	1562.56	781.28

ALT	Corresponding Diluent (mL)									
	HollisterStier (n = 27)					Stallergenes Greer (n = 5)				
	PNU (1000)	PNU (2000)	PNU (3000)	PNU (4000)	PNU (5000)	PNU (1000)	PNU (2000)	PNU (3000)	PNU (4000)	PNU (5000)
B										
Lowest value	90.00	45.00	30.00	22.50	18.00	1140.00	570.00	380.00	285.00	228.00
Highest value	410.00	205.00	136.67	102.50	82.00	1580.00	790.00	526.67	395.00	316.00
Mean	197.41	98.70	65.80	49.35	39.48	1348.00	674.00	449.33	337.00	269.60
St. deviation	66.27	33.13	22.09	16.57	13.25	156.26	78.13	52.09	39.06	31.25

^*^ALT stands for Alternaria

Corresponding PNU concentrations for a weight per volume of 1:100 and 1:200 are shown A, while corresponding diluent volumes for PNUs values from 1000 to 5000 are shown in B. These volumes represent the amount of diluent or buffer added to 1 g of allergen

## Discussion

This study compared weight per volume and PNU concentrations for birch, ragweed, dog and Alternaria, which are all non-standardized extracts in Canada. Our results show substantial variability when comparing weight per volume and PNU concentrations for both Hollister-Stier and Stallergenes Greer products. The largest variability was observed for Hollister-Stier’s ragweed extract and Stallergenes Greer’s Alternaria extract, while both companies had the smallest variability for their dog extract. The significance of our study is to not only highlight the variable potencies that exist within a sample of non-standardized extracts, but also show how these concentrations compare to actual allergen immunotherapy dosing recommendations in Canada and the United States.

There are currently only 19 standardized allergen extracts available in Canada, as most commercial extracts are non-standardized, including birch, ragweed, dog and Alternaria. The extraction process for both standardized and non-standardized products is essentially the same, with quality control measures being the primary difference [9]. Non-standardized extracts are labeled on the basis of PNU values, or the weight of the source material extracted with a given volume of extracting fluid (weight per volume). These approaches to labeling concentrations have no established relation for biologic potency, and there are no dose–response studies with non-standardized extracts [8]. Unestablished quality-control standards have large implications for safety and efficacy of immunotherapy as prescribers cannot reliably and consistently predict the response of each treatment based on the manufacturer labelled concentrations.

It is widely thought that the advantage of standardized extracts over their non-standardized counterparts is the consistency of biological activity based on established and consistent methods to determine potency. However, recent studies have found that there are significant differences in the composition and content of specific allergen levels among standardized extracts, like house-dust mite (HDM) [10–13]. Since extraction processes differ among manufacturers, standardized extracts may contain different amounts of allergens. In a recent study by Nolte et al. [14], differences in the content of Der 1 & Der 2, the major allergens of HDM, were observed despite equivalent concentration labelling. Mean Der 1 to Der 2 ratios of 20.5 and 5.2 were found for two batches of D. farinae from the same manufacturer labelled both as 10,000 AU/mL. The mean Der 1 to Der 2-ratio ranged from 0.4 to 20.5 among various manufacturers they examined. Similarly, a study by Jung et al. [15] looking at pollen chemistry showed that pollen ranges from 2.5% to 61% of protein by dry mass. Another study by Roulston et al. [16] concluded that there was a negative correlation with percentage of protein in pollen grain to pollen grain volume and mass. Furthermore, Schappi et al. [17] demonstrated that the concentration of birch protein (bet v 1) only represented 0.07% of the total pollen grain mass of 7.85 ng. These collective studies highlight the significant variability that exists in protein and allergen content compared to the labelled concentration of the allergen extract. The variability even in standardized extracts demonstrate the importance of not only focusing on developing standardized methods to ensure consistency of potency, but also stricter quality-control standards to ensure less variability in the production of the immunotherapy extracts.

The lack of consistency in allergenic potency and composition of non-standardized extracts affects clinical efficacy and has important implications for safety [14]. The AAAAI suggests maintenance doses for non-standardized extracts ranging from 3000 to 5000 PNU, or weight per volume 1:100 to 1:200: both at a volume of 0.5 mL [8]. The Canadian Society of Allergy and Clinical Immunology (CSACI) has recommended using 5000 PNU at a volume of 0.5 mL as a recommended maintenance dose [7]. The CSACI has suggested using PNU instead of weight per volume to try to simplify the process of prescribing subcutaneous immunotherapy.

**Fig. 1:**
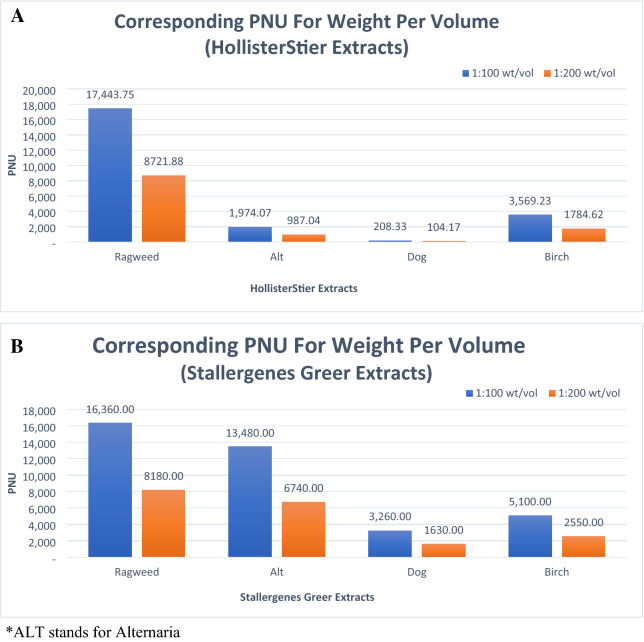
Corresponding PNU concentrations for a weight per volume of 1:100 and 1:200 are shown in Table 4A for HollisterStier extracts and in Table 4B for Stallergenes Greer extracts

From our study, the corresponding PNU concentration for a weight per volume of 1:100 to 1:200 aligns with the recommended PNU dosing for non-standardized extracts. We believe that dosing towards a weight per volume of 1:200 prioritizes safety and minimizes the risk of severe reactions with subcutaneous immunotherapy. Birch extract has low PNU concentration (below < 5000) for a weight per volume of 1:200 for both HollisterStier and Stallergenes Greer (Table 1A). In contrast, for both HollisterStier and Stallergenes Greer’s ragweed extract, a weight per volume of 1:200 corresponds to a PNU concentration greater than 5000 (Table 2A). Dog extract for a weight per volume of 1:200, and even for 1:100, corresponds to very low PNUs for both companies (Table 3A). The major allergen content for dog extract is typically too low to allow for effective dosing [8], and this is likely because the target dose of 5000 PNU may be impossible to reach based on our calculations with both companies (Table 3A). Although not part of our study, it may be possible with acetone precipitated dog extracts to reach therapeutic dosing as lower weight per volume corresponds to a higher PNU [18]. Finally, for Alternaria, corresponding PNU concentrations for HollisterStier is low at only 500 while over 5000 for Stallergenes Greer (Table 4A). The corresponding PNU concentrations for a weight per volume of 1:100 and 1:200 for all 4 extracts from both companies are summarized Table 5 and Fig. 1.

**Table 5:** Corresponding PNU concentrations for a weight per volume of 1:100 and 1:200 for HollisterStier and Stallergenes Greer extracts

RAGWEED	Corresponding PNU/mL			
	HollisterStier (n = 16)		Stallergenes Greer (n = 5)	
	Wt/volume (1:100)	Wt/volume (1:200)	Wt/volume (1:100)	Wt/volume (1:200)
Mean	17,443.75	8721.88	16,360.00	8180.00
St. Deviation	2593.98	1296.99	1209.30	604.65

ALT	HollisterStier (n = 27)		Stallergenes Greer (n = 5)	
	Wt/volume (1:100)	Wt/volume (1:200)	Wt/volume (1:100)	Wt/volume (1:200)
Mean	1974.07	987.04	13,480.00	6740.00
St. Deviation	662.68	331.34	1562.56	781.28

DOG	HollisterStier (n = 12)		Stallergenes Greer (n = 5)	
	Wt/volume (1:100)	Wt/volume (1:200)	Wt/volume (1:100)	Wt/volume (1:200)
Mean	208.33	104.17	3260.00	1630.00
St. Deviation	73.12	36.56	449.89	224.94

BIRCH	HollisterStier (n = 13)		Stallergenes Greer (n = 5)	
	Wt/volume (1:100)	Wt/volume (1:200)	Wt/volume (1:100)	Wt/volume (1:200)
Mean	3569.23	1784.62	5100.00	2550.00
St. Deviation	519.44	259.72	477.49	238.75

^*^ALT stands for Alternaria

Overall, we suggest selecting a PNU dose that corresponds to a weight per volume of 1:200 as this ensures safety to the patient. Our recommendations for starting PNU dose for the four non-standardized extracts are highlighted in Table 6. If the starting PNU concentration is considerably below 5000 for a weight per volume of 1:200 or proves to be ineffective, such as in birch or Alternaria with HollisterStier, slow up-titration is advised. Conversely, for starting PNU concentrations above 5000 that corresponds to a weight per volume of 1:200, such as ragweed or Alternaria with Stallergenes Greer, we recommend a maximum starting PNU concentration of 5000.

**Table 6:** Recommended lowest PNU concentrations for birch, ragweed, dog and Alternaria, based on a weight per volume of 1:200 for both HollisterStier and Stallergenes Greer’s extracts

	Recommended PNU/mL	
	HollisterStier	Stallergenes Greer
Birch	1500	2300
Ragweed	5000*	5000*
Dog	25	1200
Alternaria	500	5000*

If concentrations were stronger than 5000 PNU/mL for a 1:200 weight per volume, then a maximum PNU concentration of 5000 was recommended, as indicated by an asterixis (*)

Based on our findings and the relative lack of randomized controlled trial data, the efficacy and safety of dog immunotherapy is questionable. We do not recommend dog immunotherapy at this time until further data is available. However, if prescribed, we suggest starting PNU concentrations of 25 for HollisterStier and 1200 for Stallergenes Greer with slow up-titration as needed. Table 7 highlights recommended weight per volume dosing when converting from PNU concentration of 3000. Individual calculations may need to be undertaken for the various non-standardized extracts, as concentrations can vary between extract batches from within the same allergen manufacturer, and certainly between manufacturers. As such, multiple allergen immunotherapy is preferred to be ordered from the same manufacturer.

**Table 7:** Recommended lowest weight per volume concentrations for birch, ragweed, dog and Alternaria, based on a PNU concentration of 3000 for both HollisterStier and Stallergenes Greer’s extracts

	Recommended weight per volume	
	HollisterStier	Stallergenes Greer
Birch	1:150	1:200
Ragweed	1:800	1:600
Dog	1:100*	1:100
Alternaria	1:150	1:500

If concentrations were stronger than 1:100 weight per volume for a 3000 PNU/mL, then maximum weight per volume of 1:100 was recommended, as indicated by asterixis (*)

The efficacy of immunotherapy depends on achieving an optimal therapeutic dose of the allergen extract [8]. Unlike non-standardized extracts, standardized extracts have been extensively studied [19] and doses used in controlled clinical trials form the basis of the recommended dose ranges [8]. For non-standardized extracts, the therapeutically effective doses must be estimated and individualized [8]. Allergen concentrations that are too low are less likely to be effective, while those that are too high may result in systemic reactions. The variability in biological potency that is present in not only non-standardized extracts, but also standardized extracts, can potentially affect the outcomes of clinical trials trying to prove the effectiveness of allergen immunotherapy. A literature review of trials using non-standardized extracts, including those of birch, dog dander and Alternaria, revealed that extracts are not standardized between studies. Although many clinical trials attempted to individually standardize extracts within their own study, the method of standardization was variable between studies [20]. Various units were used including specific unit (SU) [21], biological unit (BU) [22], Radioallergosorbent test (RAST) [23] units, and weight of the extract [24], with most units being arbitrarily developed. The maintenance dose also varied greatly between studies, up to more than a tenfold difference at times [22, 25]. The usefulness of these collective studies is certainly limited by the different biological units used and the variability in dosing, making it difficult to extrapolate to other clinical situations. This study also does not include data from other extraction processes (natural source versus laboratory cultures), or delivery methods for allergen extracts in immunotherapy, which may reduce external validity of the results. As we do not have reliable efficacy data for non-standardized immunotherapy extracts, we believe that these extracts should be prescribed in a safer manner by aiming for a 1:200 weight per volume dose and a corresponding PNU below 5000. If this is not therapeutically effective, up-titration should be pursued.

Ultimately, based on our study, we strongly advocate for non-standardized extracts to move towards standardization to improve safety as well as efficacy. As demonstrated in our study, both safety and efficacy data of immunotherapy in many non-standardized extracts is uncertain and unpredictable given high variability of allergen potency. Although standardized extracts also have variable potencies which poses its own challenges, it is nonetheless more consistent and thereby safe compared with non-standardized extracts. Challenges exist as implementing standardization to non-standardized extracts would require high quality randomized controlled trials to support policy changes and manufacturing regulations. It would also require multiple stakeholders to be involved including clinicians, manufacturing companies, and regulatory bodies. Currently regulations regarding non-standardized allergens are minimal to non-existent.

Limitations in this study include the small sample of data, the limited number of allergens assessed, and only data from 2 allergen manufacturers were analyzed. However, we believe these trends would be similar among other non-standardized extracts and for other manufacturers. Also, major allergen levels were not available for the allergens assessed in this study.

Taken together, our study highlights the substantial variability that exists in extract quantification for four non-standardized extracts. More importantly, we observed that that allergen potencies as currently manufactured may not meet the immunotherapy dosing recommendations. From our results, we have demonstrated that the conversion to recommended doses between weight per volume and PNU is variable between lots and between companies for all of the non-standardized allergens studied. Doses of allergen extract should be therapeutically effective, while minimizing the risk of harm. Future research will be necessary to examine larger batches of extracts, and from more manufacturers. Most importantly, randomized controlled trials should be performed to identify safe and clinically effective doses for allergen immunotherapy. Until further research and clinical trials, we recommend clinicians to thoroughly evaluate biological potencies prior to use and administration in immunotherapy.

## Data Availability

Not applicable.
